# Regional Differences in Intervention Coverage and Health System Strength in Tanzania

**DOI:** 10.1371/journal.pone.0142066

**Published:** 2015-11-04

**Authors:** Claud J. Kumalija, Sriyanjit Perera, Honorati Masanja, Josibert Rubona, Yahya Ipuge, Leonard Mboera, Ahmad R. Hosseinpoor, Ties Boerma

**Affiliations:** 1 Policy and Planning Department, Ministry of Health and Social Welfare, Dar es Salaam, United Republic of Tanzania; 2 CTS Global, assigned to Centers for Disease Control and Prevention, Dar es Salaam, United Republic of Tanzania; 3 Ifakara Health Institute, Dar es Salaam, United Republic of Tanzania; 4 World Bank, Dar es Salaam, United Republic of Tanzania; 5 National Institute for Medical Research, Dar es Salaam, United Republic of Tanzania; 6 Department of Health Statistics and Information Systems, World Health Organization, Geneva, Switzerland; Leibniz Institute for Prevention Research and Epidemiology (BIPS), GERMANY

## Abstract

**Background:**

Assessments of subnational progress and performance coverage within countries should be an integral part of health sector reviews, using recent data from multiple sources on health system strength and coverage.

**Method:**

As part of the midterm review of the national health sector strategic plan of Tanzania mainland, summary measures of health system strength and coverage of interventions were developed for all 21 regions, focusing on the priority indicators of the national plan. Household surveys, health facility data and administrative databases were used to compute the regional scores.

**Findings:**

Regional Millennium Development Goal (MDG) intervention coverage, based on 19 indicators, ranged from 47% in Shinyanga in the northwest to 71% in Dar es Salaam region. Regions in the eastern half of the country have higher coverage than in the western half of mainland. The MDG coverage score is strongly positively correlated with health systems strength (r = 0.84). Controlling for socioeconomic status in a multivariate analysis has no impact on the association between the MDG coverage score and health system strength. During 1991–2010 intervention coverage improved considerably in all regions, but the absolute gap between the regions did not change during the past two decades, with a gap of 22% between the top and bottom three regions.

**Interpretation:**

The assessment of regional progress and performance in 21 regions of mainland Tanzania showed considerable inequalities in coverage and health system strength and allowed the identification of high and low-performing regions. Using summary measures derived from administrative, health facility and survey data, a subnational picture of progress and performance can be obtained for use in regular health sector reviews.

## Introduction

Regular reviews of health systems progress and performance are critical for the improvement and implementation of health policies and programmes. Such reviews should be part and parcel of the implementation of the national health sector strategic plan and focus on the analysis of progress towards national targets for the core indicators of the national plan [1].

Assessments of inequalities in health services and intervention coverage within countries should be an integral part of health sector reviews in, for instance, the context of universal health coverage strategies [2]. Multiple stratifiers can be used to track inequalities between men and women, by socioeconomic status and place of residence. Subnational differences by administrative units such as districts or regions (or provinces) are of particular interest, because they are closely linked to policy decisions about resource allocation and program targeting. Therefore, a systematic assessment of subnational health progress and system performance should be a key element of regular reviews.

Some countries regularly analyze multiple health indicators to assess the relative performance of subnational administrative units for monitoring purposes. For instance, the South Africa health barometer is produced annually using health facility reports and administrative data to assess progress in 42 indicators for 52 districts and 9 provinces [3]. In Uganda, district league tables are produced to assess performance in 111 districts using an aggregate score of management and coverage indicators [4].^4^ Research studies have also used subnational indicators to evaluate the performance of national programs that aim to reduce subnational inequalities. For instance, in Brazil data from 554 microregions were used to assess the impact of an integrated community based primary care program on infant mortality, while controlling for health determinants [5]. In Mozambique, child mortality trends derived from three household surveys showed an association with health system strength [6]. Others have used selected survey-based coverage indicators to assess subnational health systems performance [7].

This paper assesses subnational performance through measurement of health system strength and coverage of interventions, while taking socioeconomic differences into account, in the context of the midterm review of the health sector strategic plan 2009–2015 (HSSP III) in mainland Tanzania. The assessment was based on a synthesis of indicators obtained from multiple data sources, including health facility, household survey and administrative data.

## Methods

Tanzania mainland had a population of 43 million in 2012 and was, until 2012, divided into 21 administrative regions [8]. In 2012, four new regions were created to bring the total to 25. This analysis uses data for 2012 and earlier and will focus on the 21 regions. The capital region of Dar es Salaam region is the most urbanized, with about 10% of the mainland population, in a largely rural population (71% rural in 2012 census).

The implementation of HSSP III [9] is guided by annual reviews that are informed by health performance profiles and a more extensive midterm review of progress [10,11]. This study on subnational progress and performance was part of the midterm review conducted in 2013. The analytical team consisted of the Ministry of Health and Social Welfare, National Institute for Medical Research, Ifakara Health Institute and the World Health Organization.

The aim was to assess health system strength and coverage of interventions, related to the priority indicators of HSSP III, taking into account the regional levels of socioeconomic development. Coverage is defined as the proportion of people who receive a specific service or intervention among those who need the service or intervention. Composite measures were computed from a range of indicators on socioeconomic development, health system strength and coverage for health Millennium Development Goals interventions (MDG coverage score). HSSP III did not include indicators for monitoring the coverage of interventions against noncommunicable diseases.

The main data sources for the regional performance assessment are household surveys, health facility reports and administrative data. The household surveys include four Demographic and Health Surveys (DHS), two HIV and malaria indicator surveys, one HIV indicator survey and a national immunization coverage survey [12–19]. Health facility data are derived from the Ministry of Health and Social Welfare database and include annual data for the period 2009–2012. The denominators for the coverage estimates from the health facility reports are obtained from the 2012 population census, with back projections using the intercensal growth rates by region. In addition, ministerial databases on health workforce and health infrastructure are used for the measurement of current health system strength.

The regional level of socioeconomic development is computed from the Tanzania DHS 2010 based on two equally weighted components: the proportion of population living in the two poorest wealth quintiles and the mean years of education of women and men aged six years and older in households.^15^

The regional health system strength is based on indicators of health infrastructure, health workforce and service utilization and quality. No data were available on the financial resources by region. The following seven indicators were used:

- Health infrastructure:
  - Health facility density per 10,000 population: no reliable data were available on population living within a specified distance or travel time from health facilities, which would have been a preferred indicator. Health facilities include public and private hospitals, health centres and smaller clinics.
  - Inpatient beds per 10,000 population
- Health workers:
  - Doctors and non-physician clinicians per 10,000 population: in Tanzania, as in many other countries, non-physician clinicians (assistant medical officers and clinical officers) play a major role in the provision of clinical services [20];
  - Nurse-midwives per 10,000 population: includes nursing professionals, midwives and enrolled nurses;
- Service utilization and quality
  - Hospital admissions per 100 population per year;
  - Outpatient department visits per capita per year;
  - Hospital case fatality per 100 admissions: an indicator of the quality of care.

All data were available for 2012 except hospital bed density data which refer to 2011. The analysis focused on the relative health system strength of each region. Therefore, the regional value for each of the seven indicators was transformed into a z-score (standard deviations from the Tanzania mainland mean), which were averaged for each of the three components. The health workforce component was given double the weight in the overall health systems score.

Nineteen health intervention coverage indicators were used to assess the performance of the regions. For several indicators of health intervention coverage data from both surveys and facility reports were available. While the survey data are generally of higher quality than the facility data, they have considerable sampling errors for subnational data and are often not as recent as facility data. Many survey-based indicators are based on recall of events by the respondents and refer to several years prior to data collection. Where possible regional statistics based on facility data were used to obtain recent estimates. Survey data were used to externally validate the facility data for the earlier years.

Only indicators for which the population in need of the intervention could clearly be defined and measured were used. The only exception is the family planning indicator derived from the facility data based on number of visits per year. The numerator may include multiple visits by the same individual, depending on the methods of contraception, and no data were available on the number of married couples per region. Thirty family planning visits per 100 population was defined as 100% and all regional rates were computed as a proportion of this rate. This indicator was included in addition to the proportion of demand for family planning satisfied from the DHS 2010 to obtain more recent statistics for the regions.

The MDG coverage score was computed for 19 indicators grouped into seven intervention areas: family planning, antenatal care, delivery and postnatal care, child health, HIV/AIDS, tuberculosis and malaria. Table 1 summarizes the indicators and data sources and describes how the score for intervention area was computed. For all indicators a single data source was selected, aiming to use the most recent reliable data. For the coverage of antenatal care (first visit) and institutional delivery data from the national survey in 2010 (considered more reliable) and the facility data for 2011 and 2012 (more recent, but slight overestimate compared to the survey) were used to obtain a recent estimate. The indicators, and the weights for the indicators within each of the four intervention areas, were chosen based on assessment of the data availability, data quality including consistency over time, consistency between facility and survey data, plausibility of the estimates generated from the facility data, and public health relevance of the intervention. A compilation of all health statistics for each region is provided elsewhere [11].

**Table 1:** Indicators included in the Millennium Development Goals coverage score, with weighting.

	Indicator	Data source
	*Family planning (FP)*: *(a+b) /2 (weight 0*.*15)*	
a	Family planning visits per 100 population (expressed as a proportion of 30 visits per 100 population as theoretical maximum)	HMIS, 2012
b	% of women 15–49 whose need for FP is satisfied	DHS 2010
	*Antenatal care*: *((c+d+e)/3 + (f+g)/2 + h) / 3 (weight 0*.*15)*	
c	% of pregnant women who made at least one antenatal visit	DHS 2010, HMIS, 2011 & 2012
d	% of pregnant women who made their first antenatal visit before 20 weeks	HMIS 2011 & 2012
e	% of pregnant women who had tetanus protection at birth (based on life time doses)	DHS 2010
f	% of HIV positive women who were on ARVs to prevent mother to child transmission (including those on ARV therapy themselves)	NACP, 2011
g	% of pregnant women who received voluntary counseling and HIV testing	THMIS, 2011/12
h	% of pregnant women who took at least two doses of SP as intermittent preventive therapy for malaria	THMIS, 2011/12
	*Delivery and postnatal care*: (3*i + j) /4 (weight 0.*15)*	
i	% of deliveries in health institutions	DHS 2010, HMIS, 2011 & 2012
j	% of women who received a postnatal checkup within 2 months after delivery	DHS 2010
	*Child health*: ((k+l+m)/3*4 + n + o) / 6 (weight 0.*15)*	
k	% of children aged 1 year who have received BCG vaccination	HMIS, 2012
l	% of children aged 1 year who have received measles vaccination	HMIS, 2012
m	% of children aged 1 year who have received DTP3 / pentavalent vaccination	HMIS 2012
n	% of children 6–59 months who received vitamin A supplementation in the last six months	DHS 2010
o	% of children under 5 years who were registered at birth	DHS 2010
	*MDG 6 indicators (weight 0*.*10 for TB (p)*, *0*.*20 for HIV ((q+r)/2)*, *and 0*.*10 for malaria (s)*	
p	% of TB cases that were successfully treated (a quality of care indicator)	NTBLCP, 2012
q	% of adults (15–49) who have been counseled and HIV tested in the last 12 months	THMIS, 2011/12
r	% of adults in need (CD4 cell count below 350) who receive ARV therapy	NACP, 2011
s	% of children who slept under a ITN last night	THMIS, 2011/12

HMIS = health management information system (facility reports); THMIS = Tanzania Health and Malaria Indicator Survey; NACP = National AIDS Control Programme; NTPLCP = National Tuberculosis and Leprosy Control Programme

In addition, long term regional trends in maternal and child health intervention coverage were analysed using four demographic health surveys during 1991–2010, focusing on four equally weighted intervention areas: family planning, maternal and newborn health, immunization and treatment of sick children. This coverage score has been developed for and used in the Countdown 2015 for maternal, newborn and child survival for equity monitoring [21].

Performance is assessed in different ways. First, the coverage levels and trends are used to rank regions. Second, performance can be assessed in terms of coverage rates in relation to the current health system strength. Third, performance is judged as coverage achievements relative to the socioeconomic level of development. In this case, health system strength is considered an intermediate variable.

We conducted a bivariate and adjusted linear and quadratic regression analysis to examine the associations between the coverage score, health system strength and level of socioeconomic development at the regional level. All analyses were conducted in Microsoft Excel and Stata version 12.

## Results

**Fig 1:**
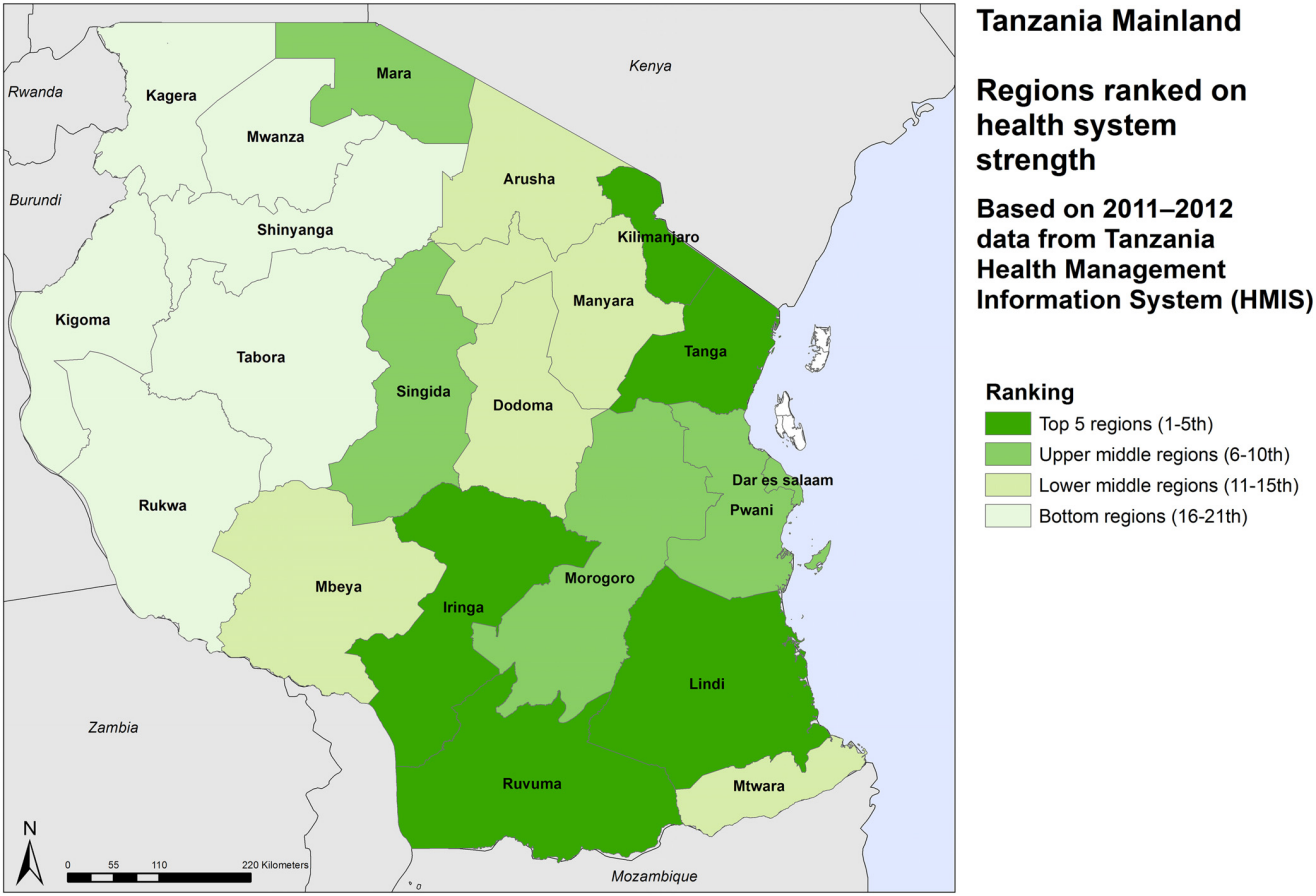
Relative ranking of regions on the health system strength, Tanzania mainland, 2012.

**Fig 2:**
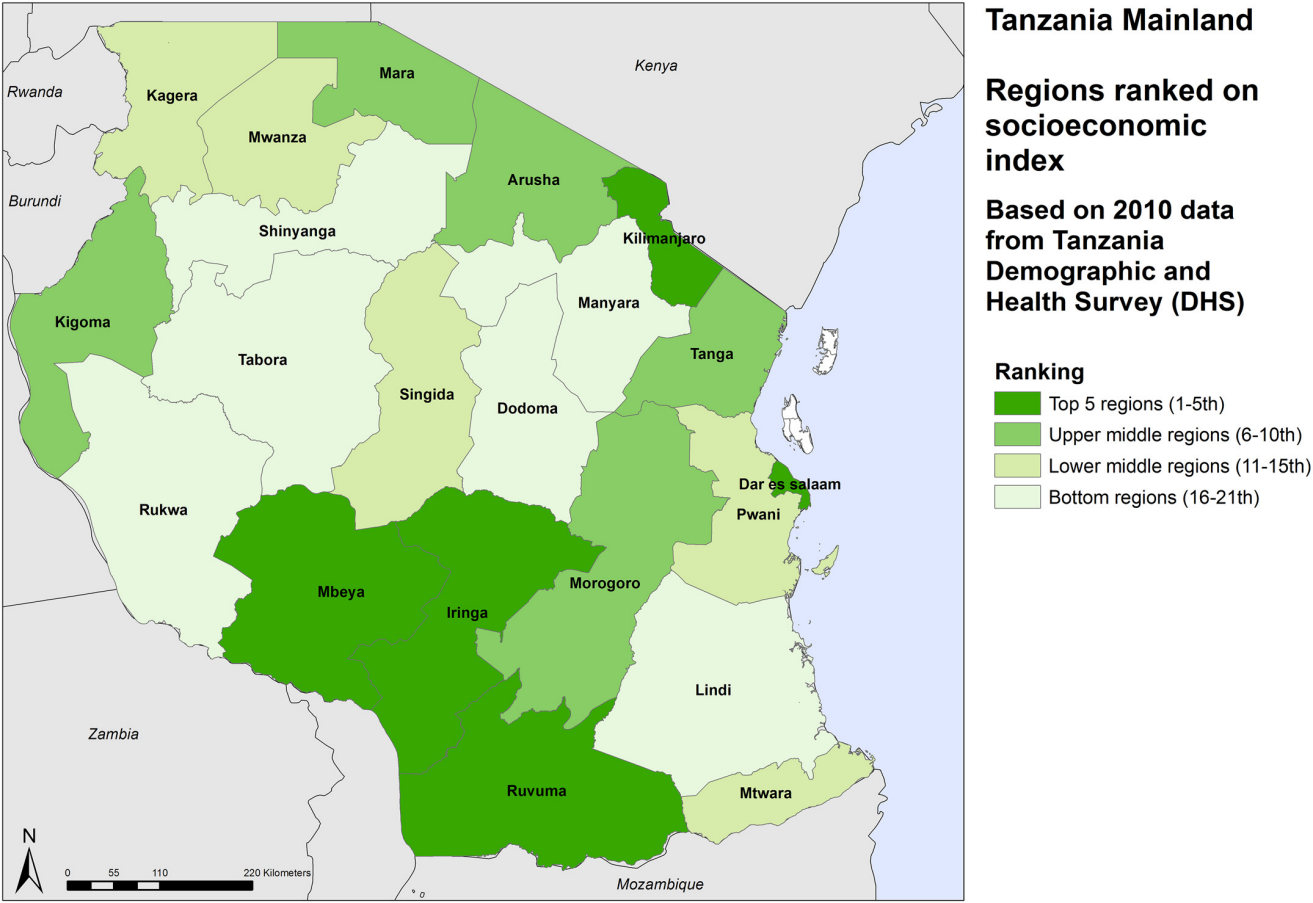
Relative ranking of regions by level of socioeconomic development, Tanzania mainland, 2012.

**Fig 3:**
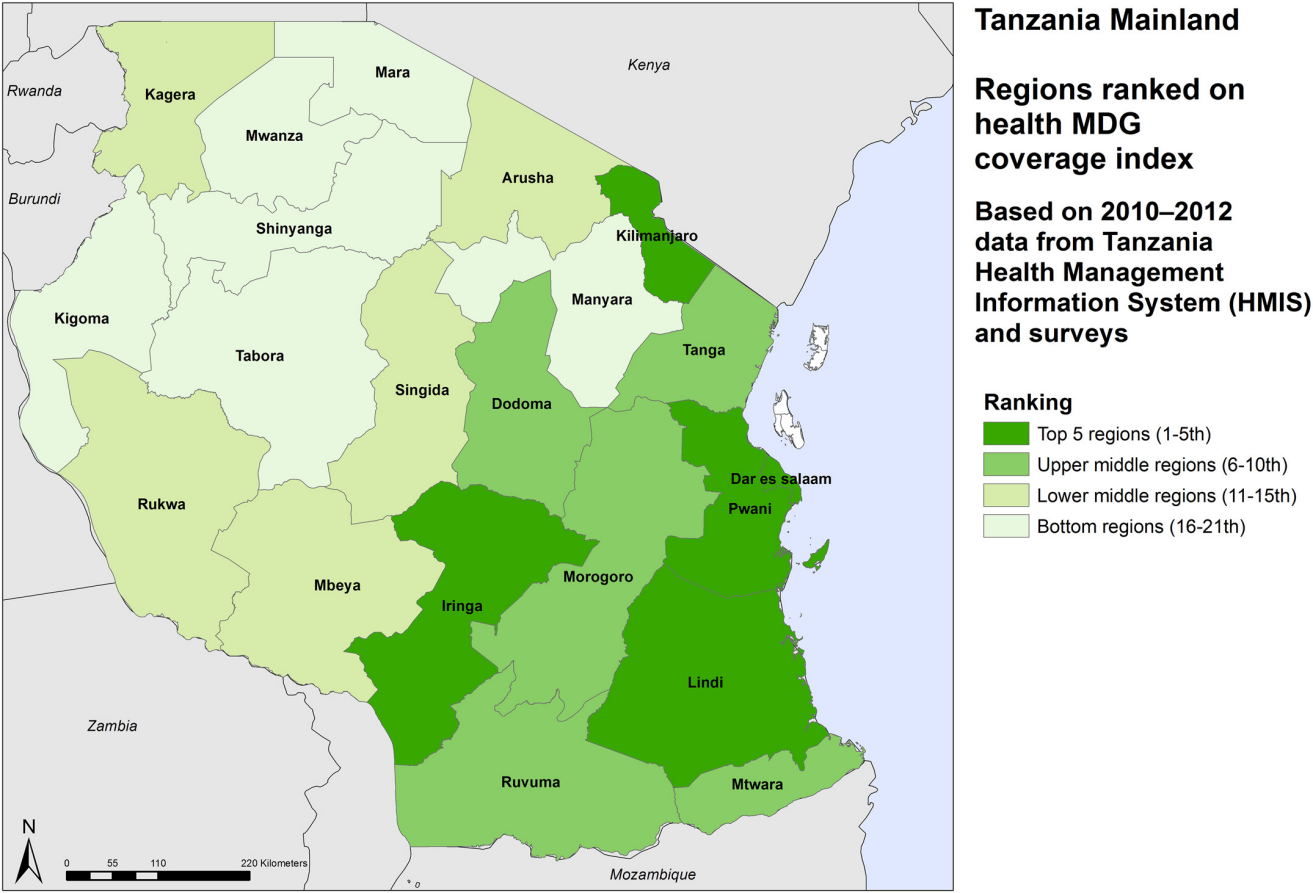
Relative ranking of regions by health MDG coverage score, Tanzania mainland, 2012.

There are large differences in the MDG intervention coverage rates. Dar es Salaam region has the highest rate (over 70%) and Shinyanga the lowest (47%) (Table 2). There is a clear pattern with the regions in the eastern half of the country having higher coverage rates than the western half of mainland (Figs 1, 2 and 3).

**Table 2:** Coverage score by region, 2010–12: ranking and scores, Tanzania mainland, 2012.

		Rankings		Score (%)			Zscore	Zscore
	MDG coverage score	Health system strength	Socio-economic status	MDG coverage	RMNCH coverage	HTM coverage	Health system strength	Socio-economic status
Dar es Salaam	1	8	1	71.1	69.0	70.7	-0.05	2.20
Pwani	2	5	12	66.7	63.4	76.0	0.35	-0.29
Kilimanjaro	3	1	2	66.3	63.1	73.4	1.38	1.79
Iringa	4	2	3	66.1	64.0	69.9	0.85	1.29
Lindi	5	3	19	65.7	61.5	78.6	0.54	-1.28
Ruvuma	6	4	4	64.4	60.7	77.9	0.53	0.94
Mtwara	7	15	15	63.0	60.3	67.9	-0.65	-0.47
Tanga	8	6	9	60.4	56.6	73.2	0.28	0.18
Morogoro	9	7	7	59.9	56.9	68.2	-0.01	0.46
Dodoma	10	11	20	58.9	55.0	69.6	-0.42	-1.37
Mbeya	11	10	5	58.5	54.6	71.8	-0.27	0.66
Singida	12	12	13	58.2	54.1	70.5	-0.43	-0.31
Arusha	13	14	6	57.3	53.0	67.9	-0.60	0.51
Kagera	14	17	14	55.3	50.9	69.1	-0.96	-0.35
Rukwa	15	19	18	53.3	49.7	64.4	-1.21	-0.99
Manyara	16	13	16	53.1	48.3	66.5	-0.59	-0.67
Tabora	17	20	21	52.9	47.6	75.5	-1.24	-1.46
Mwanza	18	16	11	52.2	46.9	74.5	-0.86	-0.16
Kigoma	19	18	10	51.6	48.0	63.4	-1.17	-0.09
Mara	20	9	8	50.2	45.3	68.9	-0.09	0.28
Shinyanga	21	21	17	47.0	42.8	62.9	-1.37	-0.88

MDG = Millennium Development goals; RMNCH = reproductive, maternal, newborn and child health; HTM = HIV, TB and malaria

In general, regions in the eastern part of the country also have stronger health systems than the regions in western and north-western Tanzania. The health system strength score is highest in Kilimanjaro region in the northeast and lowest in Shinyanga region in the northwest. Health system strength in the Dar es Salaam is well below expectations. This is likely to be due to underreporting by private facilities, which have a much larger share of the service provision than elsewhere in Tanzania, and by the national referral hospital on several of the indicators related to infrastructure, health workforce and service delivery.

The level of socioeconomic development varies considerably between the regions. As expected, the capital region of Dar es Salaam ranks top, followed by Kilimanjaro region in the north. Iringa, Ruvuma and Mbeya regions in the south and southern highlands also score higher than most regions in the western and northwestern zones of the mainland. Tabora and Dodoma in central Tanzania have the lowest levels of socioeconomic development.

**Fig 4:**
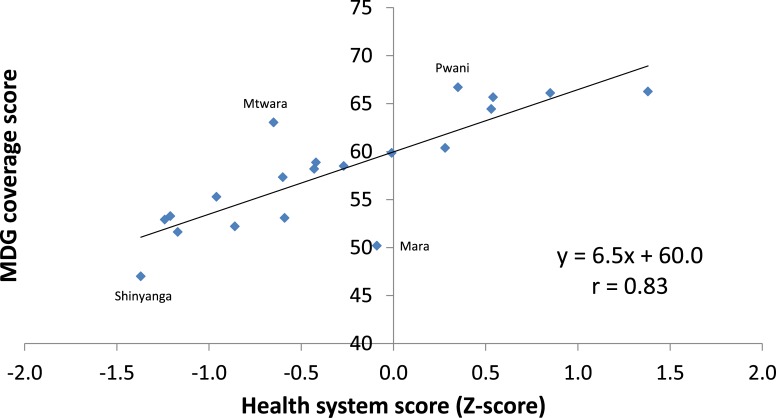
Health MDG coverage score by health system strength score, Tanzania regions.

In the analysis of the associations between the different scores Dar es Salaam was excluded, due to its unreliable data on health system strength. The MDG coverage score is strongly positively correlated with health systems strength (r = 0.83, Fig 4). The quadratic regression analysis gives a similarly good fit. A few regions lie well above the linear regression line, indicating that MDG intervention coverage is higher than expected on the basis of their health system strength. These include Mtwara in the South and Pwani in the East.

Two regions stand out as poor performers, with much lower MDG coverage than expected on the basis of their health system strength: Mara and Shinyanga in the northwest. All other regions are fairly close to the regression line: MDG intervention coverage is as expected on the basis of the strength of the health system.

The socioeconomic development score is weakly associated with coverage (r = 0.42). Controlling for socioeconomic status in a multivariate analysis has no impact on the association between the MDG coverage score and health system strength.

Table 2 also shows the rankings of the regions and permits the identification of regions which perform better in terms of coverage of services compared to socioeconomic level of development and health system strength or absolute performance.

**Fig 5:**
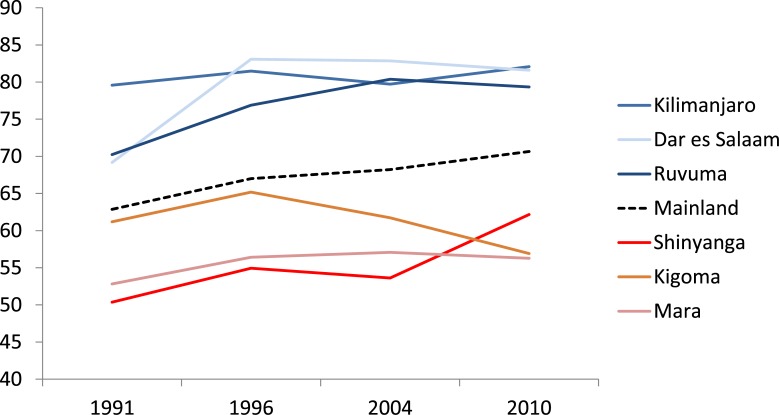
Inequalities in the Countdown MNCH coverage score (see text), based on survey data 1991–2010, national trend with three best and three poorest regions according to the 2010 survey.

The trends in the Countdown coverage score, based on four national DHS surveys during 1991–2010, show that mainland coverage improved from 59% to 70% during 1991–2010, at an annual rate of 0.6% per year (Table 3). The ranking of the regions remained fairly similar during the past two decades. Kilimanjaro, Dar es Salaam and Ruvuma regions had the highest and regions in the western part of the mainland had the lowest coverage scores throughout the past two decades. Eight regions had a relative average annual rate of increase of more than 1% during 1991–2010. Two regions did not make any progress since 1991. The absolute gap between the three best and three poorest performing regions barely changed during the past two decades and remained 22% (Fig 5).

**Table 3:** Regional trends in the Countdown maternal, newborn and child health coverage score (%), based on demographic and health survey data 1991–2010, with average annual rate of relative change of the coverage rate during the whole period (1991–2010) and between the last two surveys (2004–2010).

	Survey year				Annual rate of change (%)	
	1991	1996	2004	2010	1991–2010	2004–2010
Mainland	62.9	67.0	68.2	70.7	0.6	0.6
Kilimanjaro	79.6	81.5	79.7	82.1	0.2	0.5
Dar es Salaam	69.2	83.1	82.9	81.6	0.9	-0.3
Ruvuma	70.2	76.9	80.4	79.3	0.6	-0.2
Tanga	64.1	66.5	73.4	78.9	1.1	1.2
Mtwara	61.9	70.5	73.5	78.3	1.2	1.0
Pwani	59.7	72.6	68.1	77.7	1.4	2.2
Morogoro	64.0	69.1	76.4	77.6	1.0	0.3
Iringa	63.4	68.4	77.3	76.5	1.0	-0.2
Lindi	73.1	71.6	77.1	74.3	0.1	-0.6
Mbeya	70.3	71.5	72.0	73.3	0.2	0.3
Arusha	59.3	61.7	70.6	72.0	1.0	0.3
Singida	65.7	68.5	64.2	68.9	0.3	1.2
Kagera	53.4	62.5	70.4	68.6	1.3	-0.4
Manyara	59.3	61.7	64.9	67.5	0.7	0.7
Dodoma	64.1	65.8	68.5	67.4	0.3	-0.2
Rukwa	53.0	63.2	60.8	66.8	1.2	1.6
Tabora	65.9	72.2	55.1	62.6	-0.3	2.1
Mwanza	55.3	58.5	67.7	62.6	0.6	-1.3
Shinyanga	50.4	54.9	53.6	62.2	1.1	2.5
Kigoma	61.2	65.2	61.7	56.9	-0.4	-1.4
Mara	52.8	56.4	57.1	56.3	0.3	-0.2

## Discussion

The analysis of data from health facilities, household surveys and administrative sources provides a consistent and compelling picture of health progress and performance in the regions of Tanzania mainland. Through the creation of summary scores for coverage of MDG-related interventions, health system strength and level of socio-economic development in each of the 21 regions it is possible to obtain a comprehensive picture of inequalities between regions. Several regions had markedly different health systems strength and intervention coverage than expected on the basis of socioeconomic development. Based on the combined results of the progress and performance assessment four regions can be rated as very good (Lindi, Pwani, Mtwara, Dodoma), three as good (Iringa, Ruvuma, Kilimanjaro), four as poor (Shinyanga, Mara, Kigoma, Mwanza), and all remaining regions as average.

The effect of the health system on the MDG coverage score is considerably stronger than that of the level of socioeconomic development of the region. This indicates health systems strength is an important determinant of coverage. Poorer regions can have stronger health systems and therefore higher intervention coverage levels than expected on the basis of the level of development.

Even though considerable progress has been in made in terms of intervention coverage, major differences persist between western and eastern mainland Tanzania. These differences are for an important part due to weaker health systems, in terms health workforce and infrastructure, and to a lesser extent associated with lower levels of socioeconomic development. This strongly suggests that greater investments in health systems in most of western Tanzania and other lagging regions are needed to make greater progress in poorer performing regions.

There are several limitations of the study. This analysis did not include regional mortality trends which would provide additional information for a comprehensive performance assessment. A strong decline in child mortality levels has been observed and achievement of the 2015 MDG child mortality target of two-thirds reduction since 1990 is considered to be possible [22,23]. Regional mortality estimates of child and adult mortality in Tanzania are derived from national surveys and census. No data were available beyond 2010 and for preceding years regional estimates have large sampling errors. A second reason is that mortality trends are affected by multiple factors and our prime interest was to examine the association between health system strength and coverage trend as a more direct result of health system efforts. Other studies have however shown how multiple cross sectional surveys can provide regional estimates and be linked with trends in health system factors and coverage of interventions [5,6,10,24].

A full health systems performance assessment would also include an analysis of health system inputs, outputs in terms of service delivery and quality, as well as health impact, financial protection, and responsiveness to people’s needs [25]. Such assessments are conducted less frequently as they require considerable resources. The focus on simple measures of health system strength and intervention coverage allows a crude assessment of progress and performance at the subnational levels using data for indicators that are readily available. Data on subnational differences in intervention coverage are one of the most important pieces of health information to guide resource allocation and monitoring of progress and performance. Subnational health system performance assessment should be part and parcel of regular health sector reviews that aim to monitor progress and performance in the context of the national health sector strategic plan, using data from multiple sources that are as recent as possible.^1^

The use of a summary measure has the advantage that it captures multiple aspects of health systems and coverage. The disadvantage is that arbitrary decisions have to be made about what to include and that data availability and quality play an important role in such decisions. The indicators included in the scores used in this analysis were selected based on data availability and quality at the regional level, as well as relevance to the assessment.

Data quality is a critical issue. Tanzania’s databases on health infrastructure, health workforce, and service utilization are improving but there are still deficiencies in for instance including private sector data. Reliable long-term trend data for the health system indicators were not available. Regional hospital case fatality rates were the only indicator that measures quality of care. Some regions may have higher rates because of a concentration of tertiary hospitals that attract more complicated cases. No attempt to adjust for these risks was made.

Coverage rates that were derived from the household surveys have fairly large sampling errors at regional levels. Coverage rates that were based on health facility reports have variable quality due to either poor reporting by facilities and districts, to uncertainties in the denominator (target population), or both. An extensive assessment of the quality of all facility data including adjustments where needed preceded this study. Further investments in improving the administrative and health facility data are needed. In late 2013, an electronic reporting system of health facility data became fully operational (the District Health Information System–DHIS 2.0). Also the health workforce database is now electronic with individual level records.

From the perspective of health systems, a focus on districts is preferable above regions or provinces as this is where national resource allocations are often made. For monitoring progress and performance, however, district level data have much more “noise” in the facility data derived estimates of coverage and health system strength, and usually do not have survey data. Small area estimates can be used to fill those data gaps [26], but to-date there is little experience with such data for regular progress and performance monitoring. Regions or provinces are more suitable for such analyses as the populations tend to be larger (e.g. in Tanzania mainland on average about 2 million people) and survey-based estimates as well as health facility data are available.

The study did not include indicators on non-communicable diseases (NCD) or injuries. Tanzania’s HSSP III did not NCD targets, but this is likely to change in the next strategic plan. This would lead to including additional indicators on intervention coverage, e.g. diabetes or hypertension treatment coverage, and could also lead to more specific NCD related components in the health systems strength such as specific medicines and diagnostic tests.

## Conclusion

A combination of administrative, health facility and survey data can be used to provide recent and relevant information on subnational performance. In Tanzania, the analysis showed the existence of persistent differences in coverage of interventions between regions, which are primarily due to differences in health system strength and much less to variation in levels of socioeconomic development. Comprehensive subnational analysis of intervention coverage and health system strength based on information from multiple sources for a range of indicators should be an integral part of health sector reviews and provides a critical input into better planning and resource allocation.

## Supporting Information

S1 Table Health indicators by region for socioeconomic status, health system, coverage, and mortality. (XLSX)

S2 Table Health indicators from surveys in Tanzania, 1991, 1996, 2004/2005 and 2010. (XLSX)
